# Artificial Intelligence for Alzheimer’s Disease Diagnosis: From Traditional Machine Learning to Large Language Models

**DOI:** 10.3390/bios16090514

**Published:** 2026-09-11

**Authors:** Xiayao Guo, Yanqi Sun, Yang Chen, Hongde Liu, Xiaohui Liu, Xuemei Wang

**Affiliations:** 1 State Key Laboratory of Digital Medical Engineering, School of Biological Science and Medical Engineering, Southeast University, Nanjing 210096, China; 2 School of Computer Science and Engineering, Southeast University, Nanjing 210096, China

**Keywords:** Alzheimer’s disease, artificial intelligence, machine learning, large language models, multimodal analysis, AI-driven AD diagnosis

## Abstract

Alzheimer’s disease (AD) is the most prevalent neurodegenerative disorder and a leading cause of dementia worldwide, characterized by progressive cognitive decline, memory impairment, and functional deterioration. With the rapid growth of the aging population, AD has become a major global health challenge, imposing substantial burdens on patients, families, and healthcare systems. Despite extensive research, early and accurate diagnosis of AD remains challenging due to disease heterogeneity, overlapping clinical manifestations, and the lack of easily accessible, highly sensitive, and specific diagnostic markers. Recent advances in biomedical technologies, including neuroimaging, multi-omics profiling, electronic health records, and digital health tools, have generated large-scale and heterogeneous datasets, providing new opportunities for improving AD diagnosis. However, extracting clinically meaningful information from these complex data sources remains difficult using conventional statistical approaches. Artificial intelligence (AI) has progressively transformed AD diagnosis by evolving from traditional machine learning (ML) approaches based on handcrafted feature engineering to deep learning (DL) models capable of automated representation learning and multimodal information integration. More recently, large language models (LLMs) have further expanded the scope of AI-driven AD diagnosis by enabling contextual understanding of unstructured clinical information, knowledge-guided reasoning, and integration of multimodal biomedical evidence. This transition reflects a shift from feature-based prediction toward more flexible and intelligent diagnostic frameworks. This review synthesizes recent advances in AI-based AD diagnosis, tracing the evolution from traditional ML to DL and LLMs. Particular emphasis is placed on the emerging role of LLMs in extracting disease-related information from speech and clinical narratives, integrating heterogeneous biomedical data sources, and enabling multimodal frameworks for AD assessment.

## 1. Introduction

Alzheimer’s disease (AD) is a complex neurodegenerative disorder and the most common form of dementia, clinically characterized by memory impairment and cognitive decline [1]. With the increasing trend of aging populations, the global prevalence of AD continues to increase. It is estimated that by 2050, 150 million people will suffer from dementia [2]. The impact is expanding, posing significant burdens on patients, their families, healthcare systems, and the society [3]. Despite substantial advances in understanding AD biology, effective disease-modifying therapies remain limited, and early screening continues to represent a major challenge. Therefore, developing accurate and scalable approaches for early detection and disease monitoring has become a critical priority in AD research.

The neuropathology of AD is characterized by the deposition of amyloid-β (Aβ) peptides and abnormal phosphorylation and accumulation of tau proteins, ultimately leading to neuronal death and synaptic loss. These pathological changes may begin 20–30 years before the onset of clinical symptoms, providing a potential window for early intervention [4,5]. However, the biological heterogeneity of AD, differences in disease trajectories among individuals, and the complex interactions between genetic, molecular, structural, and functional alterations make accurate diagnosis challenging using conventional clinical assessments alone. Current diagnostic strategies, including cognitive evaluation and biomarker-based assessments, provide valuable information but may be limited by cost, accessibility, invasiveness, and incomplete representation of disease complexity.

Advances in biomedical technologies have enabled the acquisition of increasingly diverse and large-scale datasets for AD research. Neuroimaging techniques, including structural magnetic resonance imaging (sMRI), functional magnetic resonance imaging (fMRI), and positron emission tomography (PET), provide valuable information regarding brain atrophy, functional disruption, metabolic alterations, and pathological deposition [6]. Electrophysiological approaches, such as electroencephalography (EEG) and magnetoencephalography (MEG), capture dynamic changes in neuronal activity with high temporal resolution [7]. Molecular profiling approaches, including genomics, transcriptomics, proteomics, and metabolomics, reveal biological mechanisms associated with AD pathogenesis [8], whereas clinical records, electronic health records (EHRs), speech signals, and digital biomarkers provide complementary information related to cognition, behavior, and disease progression. However, these heterogeneous data sources are characterized by high dimensionality, complex interactions, noise, and substantial variability across individuals and cohorts, creating significant analytical challenges beyond the capability of conventional statistical methods.

Artificial intelligence (AI) has emerged as a powerful computational framework for addressing these challenges by enabling automated feature extraction, nonlinear pattern recognition, and integration of heterogeneous biomedical information. Traditional machine learning (ML) approaches have been widely applied for biomarker discovery and AD classification by identifying informative patterns from carefully engineered features. With the increasing availability of large-scale biomedical datasets, deep learning (DL) approaches have further advanced AD diagnosis by automatically learning hierarchical representations from imaging, molecular, clinical, and behavioral data [9,10,11]. More recently, large language models (LLMs), built upon transformer architectures and large-scale pretraining, have introduced a new paradigm by enabling contextual representation learning, knowledge integration, and flexible adaptation across diverse biomedical tasks. Beyond language-based applications, emerging multimodal LLMs have demonstrated potential for integrating imaging, clinical, electrophysiological, and other biomedical information, providing new opportunities for comprehensive disease assessment and clinical decision support [12].

In this review, we provide an overview of the of AI-driven approaches for AD assessment, emphasizing the evolution from traditional machine learning to deep learning and emerging large language model-based systems. We first summarize the major data modalities used in AD assessment, followed by a discussion of AI methods and their applications across these modalities. Particular attention is given to recent LLM-based approaches in speech and language analysis, electronic health records, multimodal assessment, and clinical information processing. We finally discuss the major challenges and future perspectives of AI-driven AD assessment.

The relevant literature was identified through searches of PubMed, Google Scholar, and arXiv for studies published between January 2016 and August 2026. The search strategy combined terms related to AD (e.g., “Alzheimer’s disease,” “mild cognitive impairment,” and “dementia”), AI (e.g., “machine learning,” “deep learning,” “large language model,” and “multimodal”), and major data modalities or applications (e.g., “neuroimaging,” “omics,” “electroencephalography,” “speech,” “electronic health records,” and “digital biomarkers”). Studies were considered eligible if they applied AI methods to AD-related assessment, prediction, classification, or clinically relevant information processing. Titles and abstracts were screened for relevance, followed by full-text assessment where appropriate. Additional relevant studies were identified through reference-list screening and citation tracking. Given the substantial heterogeneity in study populations, data modalities, AI methods, tasks, and evaluation metrics, the evidence was synthesized narratively rather than through quantitative meta-analysis.

## 2. AD Assessment via Different Data Modalities

### 2.1. Neuroimaging-Based AD Assessment

**Figure 1:**
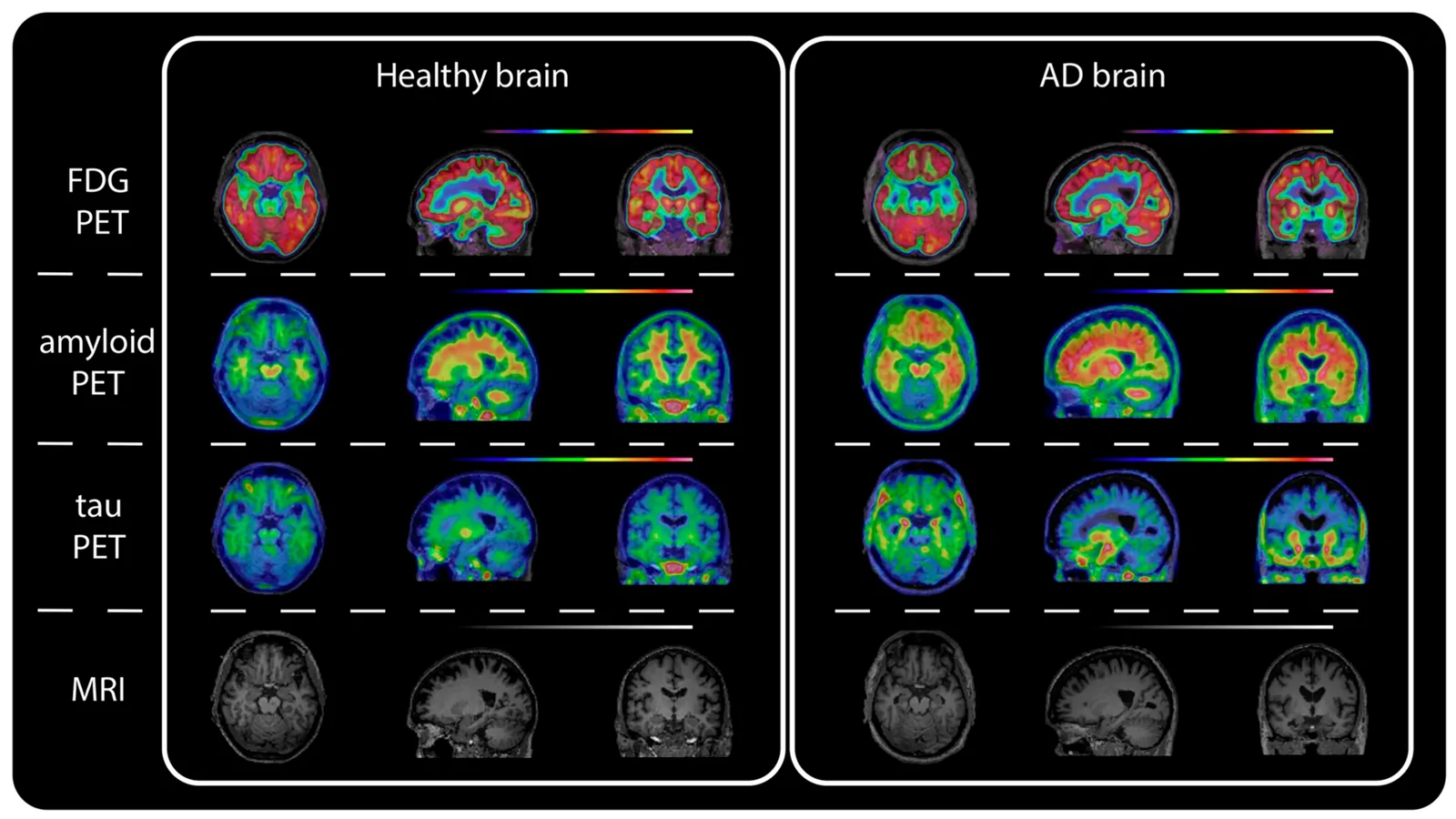
Multimodal neuroimaging features of healthy and AD brains. Representative FDG-PET, amyloid-PET, tau-PET, and sMRI images from the ADNI cohort. Compared with healthy controls, AD patients show reduced glucose metabolism, increased amyloid and tau deposition, and pronounced brain atrophy. Reproduced with permission from Ref. [18].

Neuroimaging provides a non-invasive approach for assessing brain alterations associated with AD and plays a central role in clinical diagnosis and disease monitoring. It has also become one of the most widely investigated data modalities for AI-driven AD assessment. Neuroimaging modalities used in AD research can be broadly categorized into two major types. The first type is structural imaging, such as sMRI, enabling accurate quantification of brain cortical and subcortical gray matter (GM) atrophy. Compared to cognitively intact elderly individuals, AD patients show hippocampal and entorhinal cortex atrophy on sMRI [13]. Changes in cortical thickness are markers of neurodegenerative diseases [14]. The second type is functional imaging, such as fMRI and PET. fMRI measures hemodynamic changes induced by neuronal activity, revealing specific disruptions between components of the default mode network in early AD [15]. PET is a nuclear medicine imaging technique that uses radioactive tracers to visualize and correlate molecular activity in the brain [16]. A decrease in glucose metabolism in the brain is another indicator of AD. Using 18-fluorodeoxyglucose (18F-FDG) PET, early metabolic reductions in the posterior cingulate gyrus, lateral temporal lobes, and parietal lobes of AD can be observed, which then spread to the frontal lobes as the disease progresses [17]. Amyloid PET typically uses 11C Pittsburgh Compound-B (11C-PiB) and 18-Florbetapir to demonstrate affinity for amyloid deposits. Additionally, tau PET with 18F-flortaucipir can assess aggregates of neurofibrillary tangles primarily composed of phosphorylated tau proteins. As shown in Figure 1, multimodal neuroimaging captures complementary structural, metabolic, and pathological alterations associated with AD [18].

Beyond conventional visual assessment, neuroimaging data can be further processed to generate quantitative features for AI-based AD assessment. These imaging-derived features include regional brain volumes, cortical thickness, voxel-wise intensity patterns, functional connectivity measures, and texture characteristics, which can capture subtle structural, functional, metabolic, and pathological alterations associated with AD progression. Feature extraction methods are divided into voxel-based and region of interest (ROI)-based approaches. Voxel-based methods are the simplest and most direct approaches; however, directly using voxel intensity values can lead to the curse of dimensionality and may be insufficiently sensitive to regional information, as they do not consider the spatial structure of images or capture features from specific anatomical or functional regions. ROI-based methods first define specific brain regions of interest and then extract features from each region. This approach reduces feature dimensionality and provides more interpretable features, making it widely applied in the literature. However, the accuracy of ROI definition is critical for the effectiveness of this method. Inaccurate ROI delineation or imprecise segmentation may affect subsequent feature extraction and compromise the reliability of analytical results [19]. Therefore, selecting an appropriate feature extraction strategy requires a balance between feature representation capability and anatomical accuracy to improve the stability and clinical interpretability of radiomics analysis.

### 2.2. Electrophysiological Monitoring of AD-Related Brain Dysfunction

Electrophysiological techniques provide complementary information for characterizing AD-related brain dysfunction by directly measuring neuronal electrical activity with high temporal resolution. Unlike neuroimaging modalities that primarily reflect structural, metabolic, or pathological alterations, electrophysiological recordings capture dynamic changes in neural oscillations and functional interactions among brain regions. Due to their non-invasive nature, relatively low cost, wide accessibility, and suitability for repeated measurements, electrophysiological approaches have been increasingly investigated as potential biomarkers for early detection and longitudinal monitoring of AD. The two major electrophysiological modalities applied in AD research are EEG and MEG.

**Figure 2:**
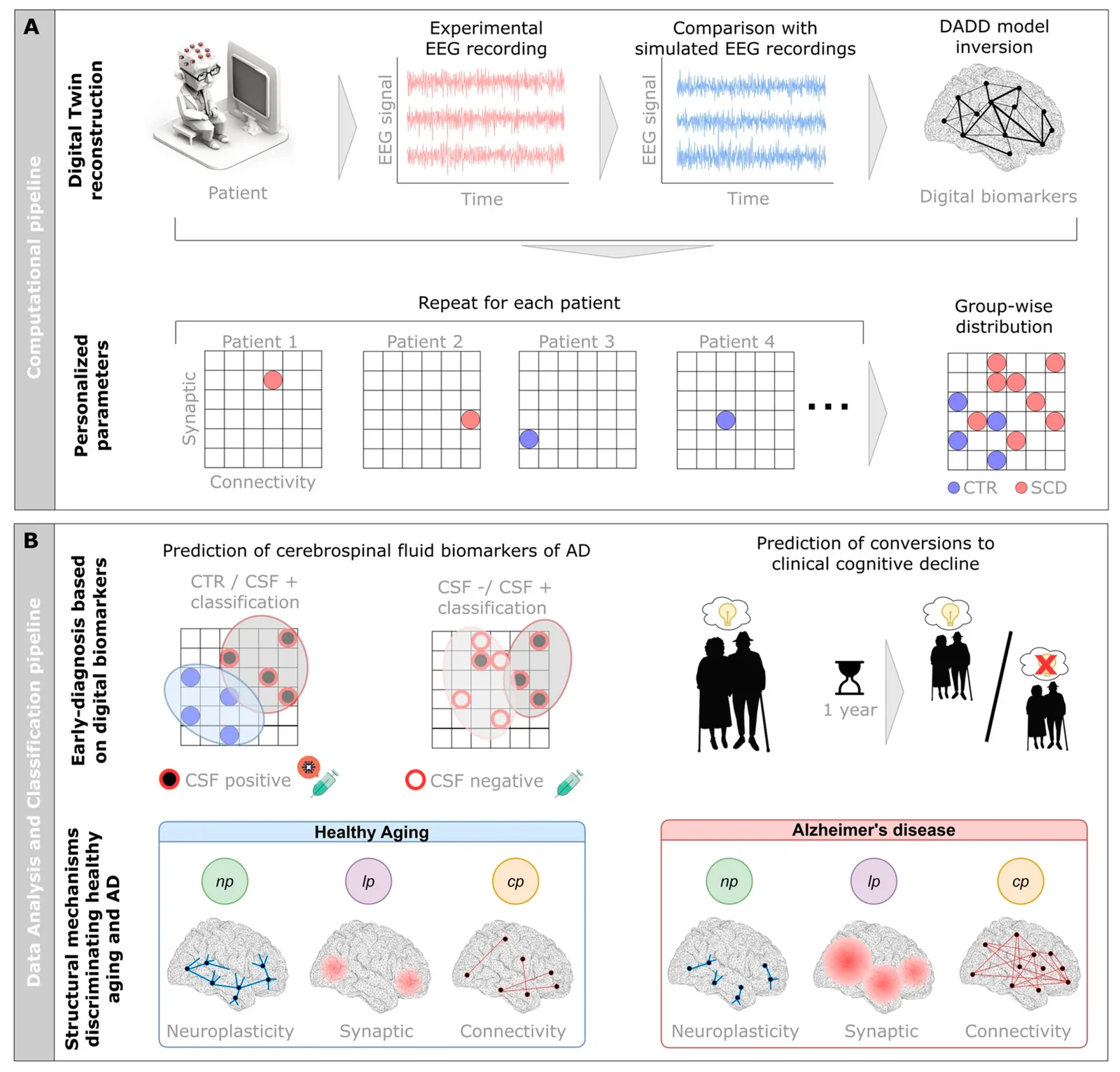
Generation of EEG-derived digital biomarkers for AD assessment. (**A**) EEG recordings are processed through computational models and personalized brain modeling approaches to reconstruct individual neurodegenerative patterns and generate subject-specific digital biomarkers. (**B**) The derived digital biomarkers reflect disease-related alterations in brain activity and connectivity, providing potential indicators for early diagnosis, pathological biomarker prediction, and monitoring of cognitive decline in AD. Reproduced with permission from Ref. [24].

EEG is the most widely investigated electrophysiological technique in AD studies due to its high temporal resolution and clinical feasibility. EEG abnormalities in AD are mainly characterized by alterations in oscillatory activity, signal complexity, and functional connectivity. A hallmark electrophysiological feature of AD is spectral slowing, characterized by increased power in low-frequency oscillations, including delta (1–4 Hz) and theta (4–8 Hz) bands, accompanied by reduced power in higher-frequency oscillations, including alpha (8–12 Hz) and beta (12–30 Hz) bands. These frequency alterations reflect disrupted neuronal synchronization and impaired functional organization of large-scale brain networks and have been associated with cognitive decline and AD progression [20]. In particular, quantitative EEG features, such as spectral power, peak frequency, and ratios between slow- and fast-frequency oscillations, have been explored as potential biomarkers for distinguishing AD from cognitively normal individuals and identifying early functional changes associated with mild cognitive impairment (MCI) [21]. Beyond spectral alterations, EEG signals also provide information regarding changes in neural complexity and functional network organization. AD-related neurodegeneration is accompanied by reduced EEG signal complexity, reflecting impaired neuronal dynamics and information processing. Therefore, complexity-based measures, including entropy-related features and nonlinear dynamic parameters, have been investigated for characterizing AD-associated alterations in brain activity [22]. In addition, EEG-derived connectivity measures, such as coherence and phase synchronization, have demonstrated disrupted functional communication between brain regions in individuals with MCI and AD [23]. These connectivity abnormalities indicate impaired coordination among distributed neural networks and provide additional electrophysiological signatures for computational analysis of AD. As shown in Figure 2, EEG recordings are processed through computational models and personalized brain modeling approaches to reconstruct individual neurodegenerative patterns, generating digital biomarkers that capture disease-related alterations in brain activity and connectivity for early AD detection and progression prediction [24].

MEG measures magnetic fields generated by neuronal electrical activity and provides high temporal resolution with improved spatial localization compared with EEG. MEG studies have identified AD-associated alterations in neural oscillations, functional connectivity, and network organization, providing additional information on disrupted neuronal communication during neurodegeneration [25]. Although its application in clinical practice is limited by higher cost and technical complexity, MEG enables more precise characterization of abnormal neuronal dynamics and complements EEG-based investigations of AD-related functional changes. Detailed review of electrophysiological signatures in AD, their relationship with disease progression, and their biomarker potential can be found in [26].

### 2.3. Multi-Omics Analysis of AD Molecular Profiles

**Figure 3:**
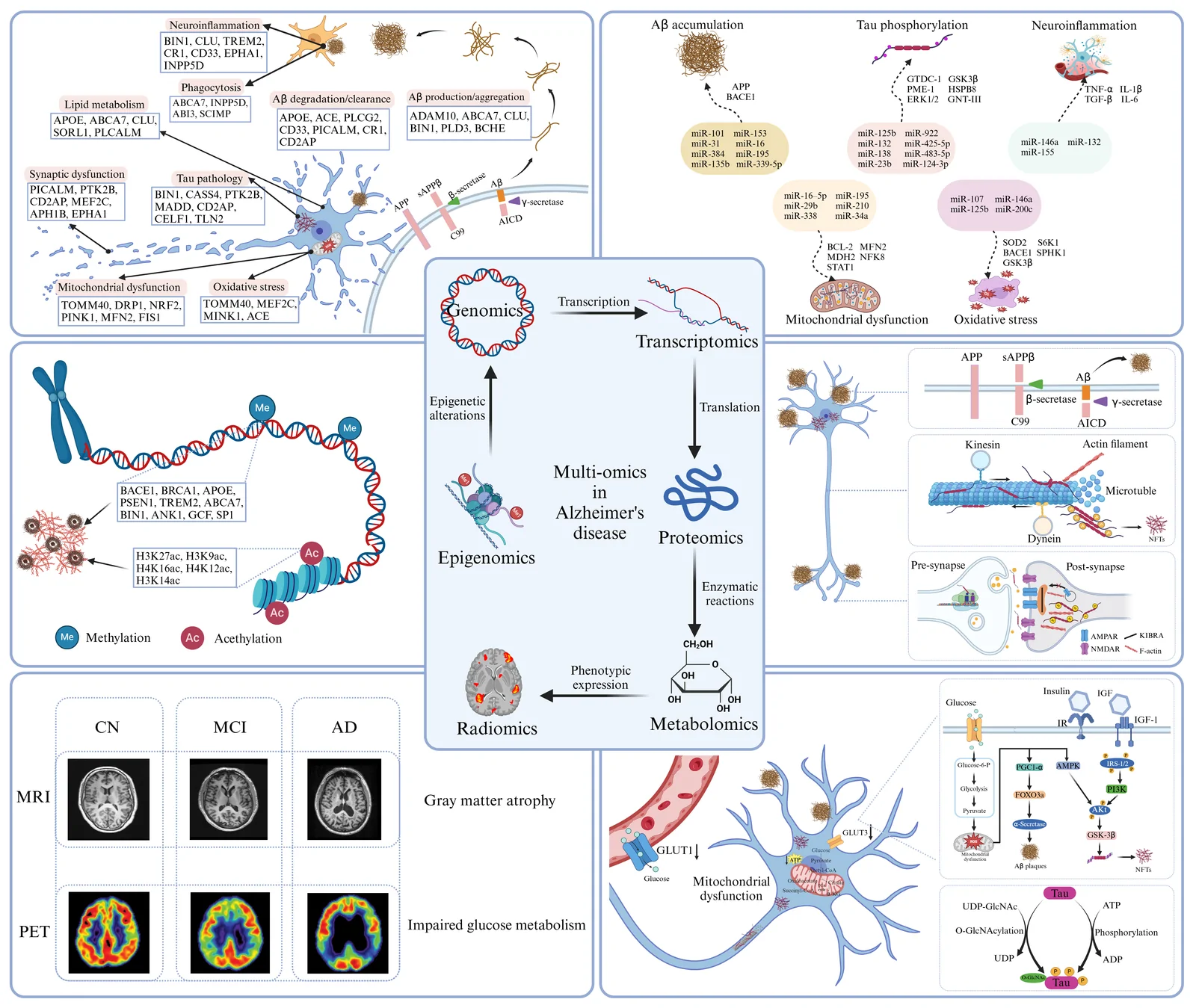
Multi-Omics insights into AD. Key pathways involved in AD are illustrated, including Aβ production/aggregation, Aβ degradation/clearance, neuroinflammation, lipid metabolism, tau pathology, mitochondrial dysfunction, synaptic dysfunction, phagocytosis, oxidative stress. Genes associated with these pathways are labeled to highlight their roles in AD pathogenesis. Dysregulation of DNA methylation and histone acetylation is associated with Aβ and tau pathology. Specific miRNAs regulate gene expression by binding to target mRNAs, influencing Aβ accumulation, tau phosphorylation, neuroinflammation, mitochondrial dysfunction, and oxidative stress. The role of protein dynamics in AD: The APP is cleaved by β-secretase into C99 and sAPPβ. C99 is further processed by γ-secretase to produce Aβ monomers and the AICD, with Aβ monomers aggregating to form amyloid plaques. Hyperphosphorylated tau disrupts axonal transport, leading to the formation of NFTs and destabilization of microtubules. In the synapses, acetylated tau inhibits the KIBRA signaling pathway, which is crucial for regulating synaptic plasticity through AMPA receptors. Glucose uptake and utilization in the brain decrease, mitochondrial dysfunction, and insulin resistance, all contributing to the exacerbation of Aβ and tau pathology in AD. Additionally, reduced O-GlcNAcylation of tau protein leads to increased tau phosphorylation. The sMRI and FDG-PET images display gray matter atrophy and reduced glucose metabolism in CN, MCI, and AD, respectively, indicating neurodegeneration and metabolic decline. Created in BioRender. Xiayao Guo. (2026) https://BioRender.com/otn9xbu.

Multi-omics technologies provide comprehensive molecular profiles of AD by characterizing biological information across multiple molecular layers, including genomics, epigenomics, transcriptomics, proteomics, and metabolomics (Figure 3).

Genomic data provide information on inherited genetic variations associated with AD susceptibility. Genome-wide association studies (GWASs) have identified numerous AD-associated genetic loci involved in diverse biological processes, including immune regulation, lipid metabolism, amyloid processing, and neuronal function, with apolipoprotein E (APOE) ε4 representing the strongest common genetic risk factor [27,28,29,30,31,32]. In addition, next-generation sequencing (NGS) approaches, including whole-exome sequencing (WES) and whole-genome sequencing (WGS), enable the identification of rare variants that may not be captured by conventional GWAS, including ARSA, CHMP2B, CSF1R, FSIP2, GRN, IGHG3, NCSTN, NOS1AP, PLD3, TM2D3, TTC3, ZBTB4, and ZNF65 [33,34,35]. Beyond conventional single-variant association testing, emerging machine learning-based approaches have been developed to enhance GWAS by leveraging high-dimensional and partially observed phenotypic data. For example, SynSurr jointly models observed phenotypes with machine learning-derived surrogate phenotypes to improve GWAS power without introducing imputation-related bias, while REGLE uses unsupervised deep learning to derive low-dimensional phenotypic representations from high-dimensional data for genetic association discovery and polygenic risk prediction [36,37]. These approaches may be particularly relevant to AD, where neuroimaging-derived and other biomarker-based surrogate phenotypes could help mitigate the limited statistical power associated with relatively few well-characterized cases.

Epigenomic data, such as DNA methylation and histone modifications, provide additional information regarding regulatory changes that influence gene expression without altering DNA sequences [38]. Epigenome-wide association studies (EWASs) have identified differentially methylated sites in AD-associated genes, such as hypermethylation of the BDNF promoter, which predicts conversion from MCI to AD, and methylation changes in PICALM and ABCA7 that correlate with Aβ pathology and cognitive decline [39]. Histone modifications, including H3K27ac and H3K9ac, have also been implicated in the dysregulation of genes involved in Aβ and tau pathology, further supporting the role of epigenetic mechanisms in AD pathogenesis [40,41].

Transcriptomic profiling using microarrays or RNA sequencing (RNA-seq) enables genome-wide quantification of gene expression changes in AD-affected tissues. Differential expression analyses have revealed dysregulated genes involved in immune signaling, synaptic function, and mitochondrial metabolism [42]. In addition to bulk transcriptomics, non-coding RNAs, including microRNAs (miRNAs) and long non-coding RNAs (lncRNAs), have been investigated as potential molecular signatures for AD detection [43,44]. Furthermore, single-cell RNA sequencing (scRNA-seq) has emerged as a revolutionary technology in biological and medical research, providing unprecedented resolution for analyzing cellular heterogeneity and transcriptomic profiles at the single-cell level [45,46,47].

While transcriptomics reflects gene expression potential, proteomics directly measures protein abundance, modifications, and interactions, providing a more functional readout of cellular states. Mass spectrometry (MS)-based proteomic analyses of cerebrospinal fluid (CSF) and blood have identified promising protein biomarkers for AD, including phosphorylated tau (p-tau181, p-tau217), neurofilament light chain (NfL), and various inflammatory and synaptic proteins [48,49]. Post-translational modifications (PTMs), such as phosphorylation, acetylation, and ubiquitination, play critical roles in regulating Aβ aggregation, tau pathology, and neuronal dysfunction, underscoring the importance of proteomic characterization for understanding AD mechanisms [50].

Cell metabolites are the end products of cellular processes, acting as the closest bridge to the phenotype. In AD, metabolomic studies have revealed dysregulation in energy metabolism, amino acid metabolism, and lipid homeostasis, reflecting mitochondrial dysfunction, insulin resistance, and neuroinflammation [51,52,53]. Reduced glucose uptake and utilization in the brain, mitochondrial dysfunction, and insulin resistance can all exacerbate Aβ and tau pathology in AD. Lipidomics, a subfield of metabolomics, has identified alterations in sphingolipids, phospholipids, and cholesterol metabolism that correlate with AD pathology and cognitive impairment [54]. Importantly, since metabolic pathways are generally conserved across species, metabolomics can also enhance the translation of preclinical studies conducted in AD animal models to humans [55].

**Figure 4:**
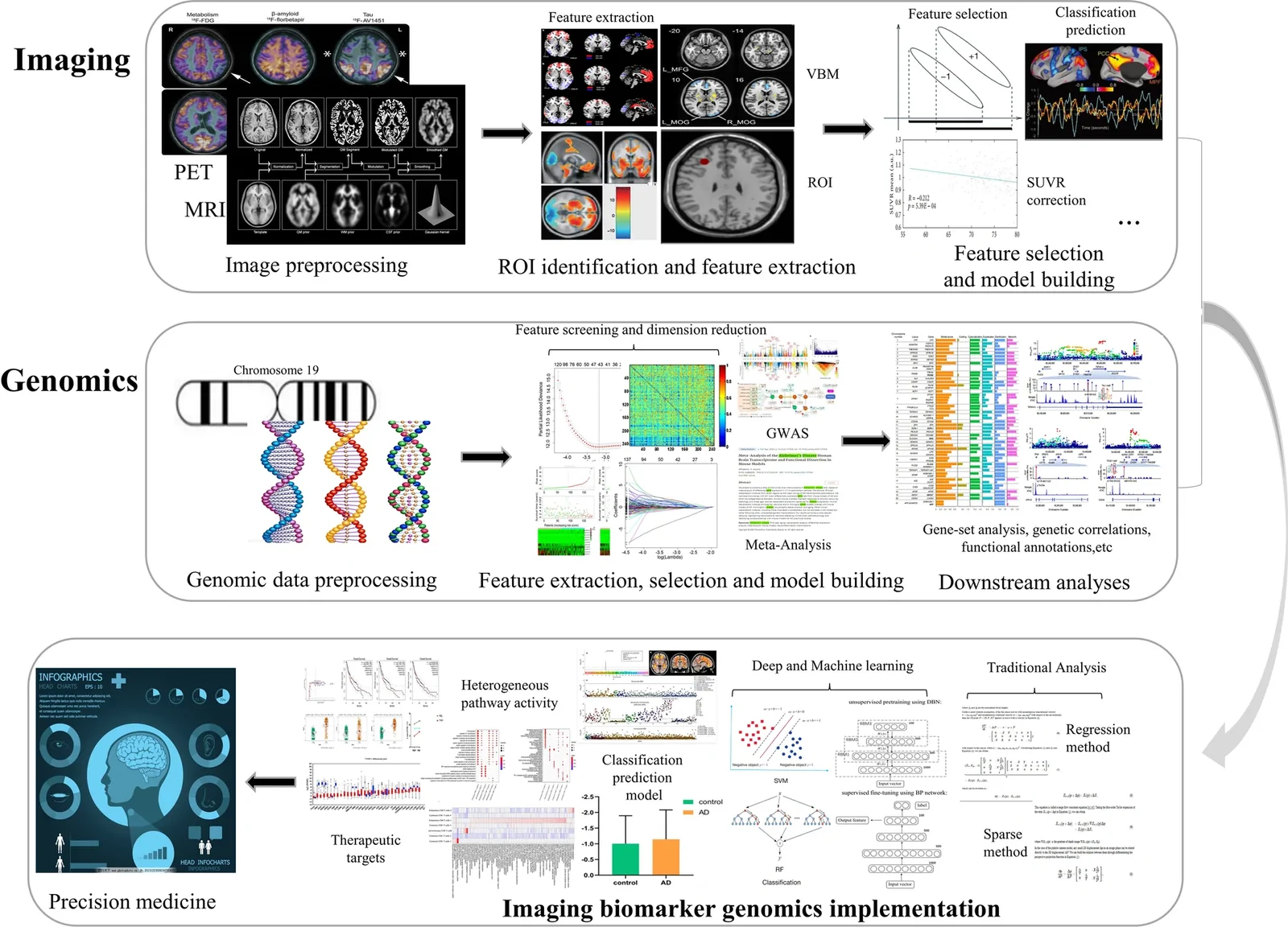
Computational framework for integrating neuroimaging and genomics data in Alzheimer’s disease studies. The framework summarizes key steps in imaging and genomic data processing, including preprocessing, feature extraction and selection, model development, and integrated analyses for biomarker discovery, classification, and prediction. Reproduced with permission from Ref. [64].

Notably, the neuroimaging modalities discussed in Section 2.1 are increasingly analyzed within the framework of radiomics, which employs quantitative image feature extraction to associate multimodal imaging characteristics with clinical outcomes. When radiomics is further integrated with genomic data, the field extends into radiogenomics, which aims to establish imaging phenotypes as surrogate, non-invasive biomarkers of underlying molecular alterations (Figure 4). Multiple studies have analyzed the associations between GWAS-validated AD risk variants and brain amyloid deposition, tau deposition, atrophy, and hypometabolism [56,57,58]. Beyond imaging–genomic integration, other cross-omics strategies have also been developed to characterize AD-related biological changes. For example, integrating transcriptomic and proteomic profiles can help reveal regulatory relationships between gene expression and protein abundance, while combined proteomic and metabolomic analyses provide insights into downstream molecular alterations beyond transcriptional regulation [59,60,61]. In addition, microbiome profiling has emerged as another molecular layer associated with AD-related biological processes through the gut–brain axis [62,63]. Compared with single-omics approaches, multi-omics integration captures biological alterations across multiple molecular layers, from genetic susceptibility to downstream functional consequences, providing a more comprehensive representation of AD heterogeneity and generating rich data resources for AI-based diagnosis.

### 2.4. Clinical and EHR Assessments for AD Diagnosis

Clinical data represent an essential source of information for AD diagnosis, encompassing diverse aspects of individual health status, including demographic characteristics, medical history, neurological examination findings, cognitive assessments, laboratory measurements, medication records, and clinical observations. Traditional AD diagnosis relies heavily on clinical evaluation, integrating patient-reported symptoms, functional decline, and standardized cognitive assessments, such as the Mini-Mental State Examination (MMSE), Montreal Cognitive Assessment (MoCA), Alzheimer’s Disease Assessment Scale (ADAS), and Brief Cognitive Rating Scale (BCRS). Although these assessments remain fundamental in clinical practice, their sensitivity can be limited during the early stages of AD, when pathological changes may precede detectable clinical symptoms. With the increasing digitalization of healthcare systems, these clinical data are increasingly captured and stored in EHRs, enabling large-scale analysis of longitudinal patient information. EHRs aggregate longitudinal patient data collected during routine clinical care, offering a rich and multidimensional view of individual health trajectories over time. For AD research, EHRs provide several distinct advantages. First, they capture real-world clinical practice data from diverse patient populations, complementing the highly controlled but often unrepresentative cohorts enrolled in clinical trials. Second, the longitudinal nature of EHRs enables tracking of prodromal signs and subtle cognitive changes that may precede formal diagnosis by years, facilitating retrospective identification of early risk indicators. Third, EHRs contain structured data elements, including diagnostic codes (e.g., ICD-9/ICD-10), laboratory test results, medication prescriptions, and vital signs, which can be systematically extracted and analyzed. Fourth, EHRs increasingly incorporate unstructured clinical narratives, including history and physical examination notes, neurology consultation reports, radiology interpretations, and discharge summaries, providing rich phenotypic information beyond structured fields [65].

Despite their value, clinical and EHR data present several challenges. Clinical records are often heterogeneous and affected by missing information, inconsistent documentation practices, variations across healthcare institutions, and differences in diagnostic procedures. Furthermore, diagnosis codes may not always accurately represent underlying disease status, and clinical notes contain complex language patterns, medical terminology, and variable documentation styles. Privacy concerns and limited data accessibility also restrict the sharing and integration of EHR datasets across institutions. Nevertheless, the multidimensional and longitudinal characteristics of clinical and EHR data provide valuable complementary information to neuroimaging and molecular datasets, and their increasing availability has created new opportunities for AI-based approaches to extract clinically meaningful patterns for AD diagnosis.

### 2.5. Speech, Language and Digital Data Analysis for AD-Related Impairment

Unlike neuroimaging or molecular biomarkers that require specialized equipment and invasive procedures, speech and digital data can be collected repeatedly in naturalistic settings with minimal burden on patients, making them attractive candidates for large-scale deployment in both clinical and community environments.

Speech and language abnormalities are among early and most prominent clinical manifestations of AD, reflecting the progressive involvement of cortical regions critical for linguistic processing. Speech and language data provide complementary perspectives: speech primarily captures acoustic, prosodic, and temporal characteristics of vocal production, whereas language reflects the cognitive processes involved in lexical retrieval, semantic representation, syntax, and discourse organization. At the speech level, AD-related alterations are commonly observed in temporal and acoustic characteristics. Individuals with early AD may exhibit reduced speech rate, slower articulation, increased pause frequency and duration, prolonged hesitation, and decreased verbal fluency, partly due to difficulties in word retrieval and cognitive planning. These temporal abnormalities can appear even during MCI, suggesting that speech production changes may precede clinically evident dementia [66,67]. At the language level, cognitive decline in AD is reflected by progressive impairments in lexical access, semantic processing, and discourse construction. Word-finding difficulty is among the earliest and most characteristic linguistic symptoms, resulting from impaired access to semantic knowledge and lexical representations. Patients with AD often demonstrate reduced vocabulary diversity, increased use of nonspecific expressions, semantic errors, repetitive words or concepts, and decreased information density. At the discourse level, impaired narrative coherence, reduced ability to maintain relevant information, and disrupted communication structure have also been reported, reflecting broader deficits in memory and executive function. Speech and language data are typically collected through structured or semi-structured tasks. Spontaneous speech tasks, such as picture description, conversational interviews, and autobiographical narration, allow assessment of natural communication patterns and provide information across acoustic, linguistic, and discourse domains. Verbal fluency tasks, including semantic verbal fluency and phonemic verbal fluency, are widely used to evaluate semantic memory retrieval and executive function, with semantic fluency being particularly sensitive to AD-related impairment [68,69,70]. These recordings can be transcribed into text, enabling integrated analysis of both speech signals and linguistic content.

**Figure 5:**
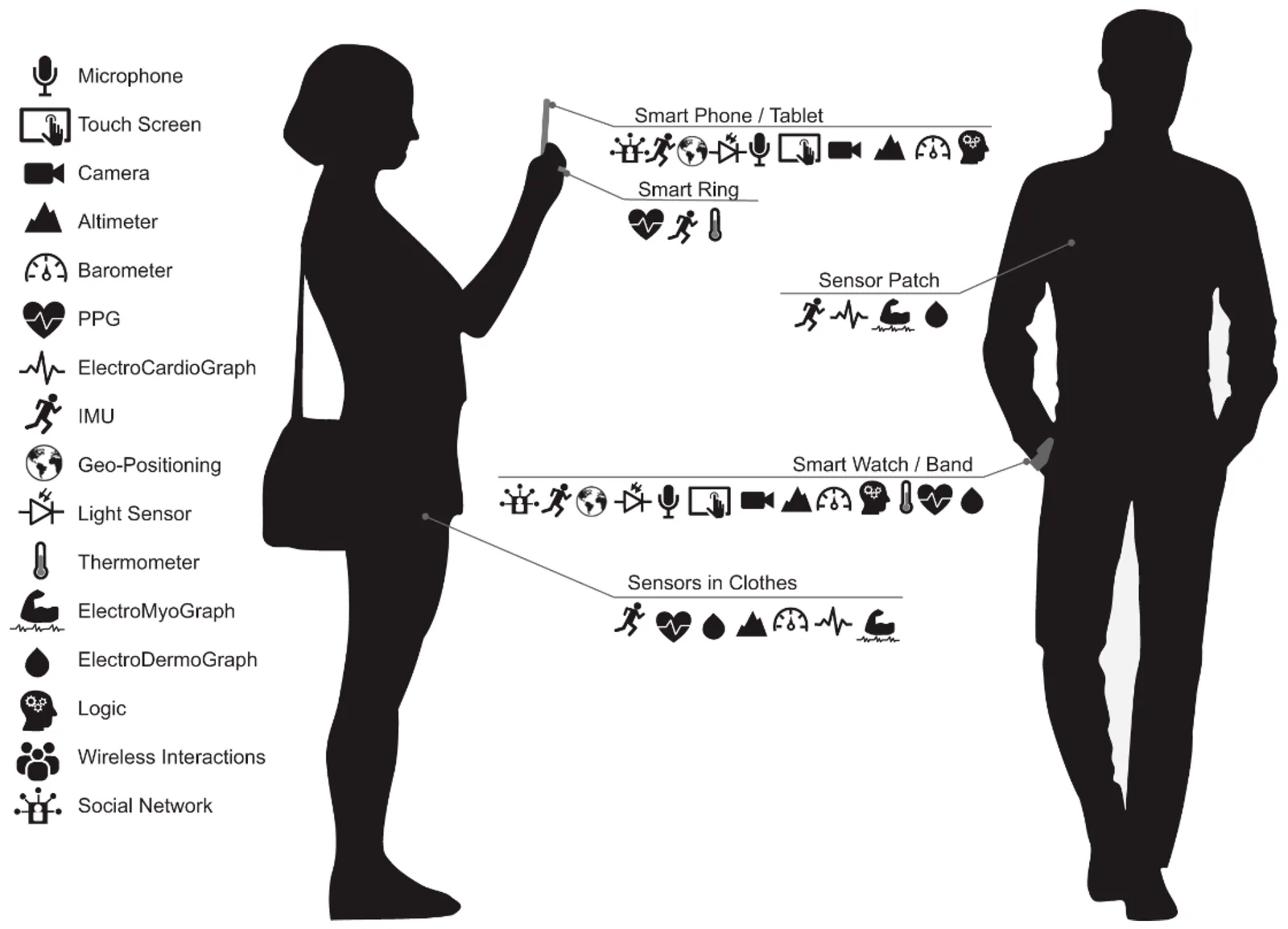
Digital data sources for AD assessment. Consumer-grade mobile and wearable devices enable continuous acquisition of multidimensional digital biomarkers, including movement, physiological, behavioral, and cognitive signals through sensors such as IMUs, PPG, microphones, cameras, and location sensors. These longitudinal digital phenotypes provide complementary information for AI-driven detection and monitoring of AD. Reproduced with permission from Ref. [82].

Beyond speech and language analysis, advances in digital health technologies have enabled the collection of multidimensional digital biomarkers for continuous and real-world assessment of AD-related changes. Digital biomarkers are objective and quantifiable measures acquired through digital technologies, including smartphones, wearable devices, and ambient sensing systems, which capture alterations in cognition, behavior, motor function, and physiology. A recent review by Nerrise et al. comprehensively summarized digital biomarkers in neurodegenerative diseases, emphasizing their classification according to sensing modalities, health domains, and clinical applications [71]. Compared with conventional clinical assessments that provide episodic measurements, digital biomarkers enable frequent, low-burden, and longitudinal monitoring, offering new opportunities for early detection and disease progression tracking in AD. Mobile and wearable devices represent major sources of digital data due to their widespread availability, connectivity, and integration of multiple onboard sensors (Figure 5). For example, inertial measurement unit (IMU)- and GPS-derived signals can characterize gait, mobility, and activity patterns, whereas photoplethysmography (PPG)-based sensors provide physiological measures such as heart rate variability and sleep-related parameters [72,73,74,75]. In addition, smartphone-derived behavioral data, including usage patterns and mobility trajectories, may reveal subtle alterations in executive function, social interaction, and daily activities associated with cognitive decline. Sensing technologies can also capture oculomotor and visuomotor alterations associated with AD. Cameras, light sensors, and dedicated eye-tracking systems can quantify eye movements, saccades, pupil responses, and visuomotor performance. Early alterations in eye movements have been reported in AD and MCI, supporting the potential value of oculomotor measures as digital biomarkers [76]. Head-mounted and wearable sensing systems can further combine eye tracking with other behavioral and motor measurements, enabling multidimensional assessment during cognitive or functional tasks. These approaches extend digital biomarker collection beyond conventional questionnaires and clinic-based examinations toward objective measurement of behavior and function in more ecologically relevant settings.

In parallel, purpose-built biosensing platforms have been developed for quantitative detection of AD biomarkers in biofluids. Biosensors generally convert specific biological interactions into measurable electrical, optical, or other physical signals and can therefore complement conventional laboratory-based biomarker assays. Field-effect transistor (FET)-based biosensors combined with machine learning have achieved high classification accuracy for core AD biomarkers including Aβ peptides, tau proteins, and neurofilament light chain in blood and serum [77,78]. Optical biosensing platforms, including surface-enhanced Raman spectroscopy (SERS) and multiplex fluorescent immunosensors, have also demonstrated promising classification performance in clinical cohorts [79,80,81]. The integration of these sensor modalities with machine learning enables scalable and non-invasive AD monitoring, complementing traditional neuroimaging and cerebrospinal fluid analyses.

## 3. AI-Driven AD Diagnosis: From Machine Learning to Multimodal Deep Learning

Compared with traditional statistical approaches, AI models can capture nonlinear relationships among complex biological features and identify subtle patterns associated with disease onset and progression. Early AI-based approaches primarily relied on conventional ML algorithms, which required handcrafted feature extraction and statistical learning to classify disease states or predict clinical outcomes. With the increasing availability of large-scale neuroimaging, molecular profiling, and longitudinal clinical datasets, DL approaches have emerged as powerful alternatives due to their ability to automatically learn hierarchical representations from raw or minimally processed data. More recently, multimodal deep learning frameworks have enabled the integration of complementary information across different data modalities, providing new opportunities for comprehensive AD detection and prognosis prediction.

### 3.1. Traditional Machine Learning-Based Diagnostic Models

Traditional ML approaches represent the earliest and most widely adopted AI strategies for AD analysis. These methods typically rely on handcrafted feature extraction, feature selection, and statistical learning algorithms to identify associations between input variables and clinical outcomes. Compared with deep learning models, classical ML approaches require relatively smaller datasets, provide more straightforward interpretability, and allow the identification of specific features associated with disease status, making them particularly suitable for biomedical studies with limited sample sizes.

In AD research, ML algorithms have been applied to diverse data modalities, including neuroimaging, molecular biomarkers, electrophysiological signals, clinical records, and digital behavioral data. Representative applications of traditional ML approaches across different data modalities, datasets, and tasks are summarized in Table 1.

Common preprocessing strategies include dimensionality reduction and feature selection methods, such as principal component analysis (PCA), LASSO, and recursive feature elimination (RFE), which are frequently used to address the high dimensionality of biomedical datasets. Supervised learning algorithms, including support vector machines (SVMs), random forests (RF), logistic regression (LR), and gradient boosting methods, have been widely applied for distinguishing CN, MCI, and AD groups, predicting MCI conversion, and identifying potential biomarkers. Among these approaches, SVM has been extensively used for high-dimensional neuroimaging and molecular datasets due to its ability to model nonlinear relationships. Ensemble methods, such as RF and gradient boosting, are advantageous for heterogeneous clinical and biological data because of their robustness and feature importance estimation capability. Regularized regression models, including LASSO and elastic net, are particularly valuable for biomarker discovery by selecting informative variables while reducing overfitting [83,84,85,86]. Beyond supervised classification, unsupervised ML methods, such as clustering approaches, have contributed to the identification of AD subtypes and disease heterogeneity [87,88].

Despite their successful applications, classical ML methods largely depend on manually engineered features and may have limited ability to capture complex nonlinear interactions among heterogeneous data modalities. These limitations have encouraged the development of deep learning approaches that can automatically learn high-level representations from large-scale biomedical datasets.

**Table 1:** Summary of representative traditional machine learning-based studies for Alzheimer’s disease.

Study	Task	Dataset	Input	Method	Result			
					Acc	Auc	Sen	Spe
[89]	CN vs. AD	ADNI (CN: 135; sMCI: 132; pMCI: 95; AD: 65)	sMRI	SVM	96.5	99.5	93.85	97.78
	CN vs. pMCI				88.7	92.8	83.16	92.59
	sMCI vs. pMCI				85.9	89.2	78.95	90.91
[90]	CN vs. AD	OASIS (CN: 254; AD: 162)	sMRI	SVM	91.3	96	-	-
				RF	92.1	96		
				KNN	90.7	97		
[91]	CN vs. AD	ADNI (CN: 111; AD: 94)	FDG-PET	SVM	-	94.5	84	95
	sMCI vs. pMCI	ADNI (sMCI: 186; pMCI: 55)			-	-	62.5	90
[92]	CN vs. AD	ADNI (CN: 36; AD: 25)	fMRI	RSVM	94.4	-	-	-
[93]	CN vs. AD	Private (CN: 120; AD: 175)	EEG	SVM	95	97	95	96
[94]	sMCI vs. pMCI	ADRC (CN: 11; sMCI: 10; pMCI: 15)	EEG	SVM			80	95
[95]	CN vs. AD	ADNI (CN: 371; AD: 267)	Genomic	SVM	-	61.4	-	-
				RF		67.4		
				LASSO		68.5		
				KNN		68.9		
[96]	CN vs. AD	MHRC (CN: 39; AD: 37)	Blood Plasma Proteins	RF	78.9	92.5	74.4	83.6
				XGBoost	77.8	89.1	76.9	79.3
				Adaboost	80	92.6	79.4	81.3
[97]	CN vs. MCI	Private (CN: 25; MCI: 25; mAD: 25)	Acoustic signal	SVM	80	-	-	75.9
	CN vs. mAD				86			87.5
	MCI vs. mAD				80			85.7
[98]	CN vs. MCI	Private (CN: 36; MCI: AD)	Acoustic signal	RF	75	67.6	81.3	66.7
				SVM	65.5	73.4	75	52.8
[81]	CN vs. AD	Private (CN: 12; AD:12)	SERS spectra	PCA-LDA	95.8	97	100	92
				PCA-SVM	91.7	97	92	92
				PCA-KNN	91.7	92	92	92
[79]	CN vs. AD	Private (CN: 3376; AD:351)	SERS spectra	SVM	98	98	99	98
				RF	96	96	96	98
				XGBoost	93	93	93	93
				CatBoost	94	94	93	95
[99]	CN vs. MCI	Private (CN: 71; MCI: 41; AD: 21)	Olfactory-stimulated fNIRS	Ensemble Learning (XGBoost + GB + LightGBM + AdaBoost)	-	86	88.7	81.8
	CN vs. AD				-	92.5	88.1	80

### 3.2. Deep Learning-Based Models

DL employs neural networks (NNs) consisting of multiple layers of nonlinear modules and backpropagation algorithms to achieve multi-level feature representation of the data, transforming minimally processed inputs into higher and more abstract representations. It does not rely on prior knowledge, instead, it guides feature extraction based on gradients of performance metrics. This has enabled DL to achieve great success in various fields, including image recognition, speech recognition, and natural language processing [100,101,102]. Recently, DL has been used to predict non-coding variants, DEGs, and to design CRISPR guide RNA, further demonstrating its potential in genetics [103,104,105]. Zhou et al. summarized the latest state of DL research related to various aspects of AD research [106].

Convolutional Neural Networks (CNNs), inspired by human visual perception, significantly improve the performance of medical image recognition by stacking multiple convolutional layers, pooling layers, and fully connected layers to progressively extract features from images, and have been successfully applied to identify AD patients using MRI images [107]. Beyond static imaging data, recurrent neural networks (RNNs) and their variants, including long short-term memory (LSTM) networks and gated recurrent units (GRUs), have been developed to model temporal dependencies in longitudinal AD-related data. These architectures are particularly suitable for capturing dynamic disease trajectories from repeated cognitive assessments, longitudinal neuroimaging measurements, electronic health records (EHRs), and digital behavioral signals. For example, LSTM-based models have been employed to predict MCI progression by learning temporal patterns from longitudinal clinical and imaging features [108]. In addition, recurrent architectures have been applied to speech and language analysis, where sequential characteristics of acoustic signals and linguistic structures provide valuable information for detecting cognitive impairment. Compared with traditional approaches based on handcrafted speech features, DL-based sequence models can automatically extract higher-level representations related to speech fluency, semantic coherence, and syntactic complexity.

Beyond conventional neural architectures, graph neural networks (GNNs) have recently gained increasing attention in multi-omics research due to their ability to model complex biological relationships represented as graphs. Unlike CNNs that primarily capture grid-like spatial information, GNNs directly operate on graph structures, allowing incorporation of prior biological knowledge, including gene regulatory networks, protein–protein interaction networks, metabolic pathways, and cell–cell communication networks. By propagating information between connected nodes, GNNs can identify biologically meaningful patterns and improve interpretability in multi-omics analysis [109,110,111,112]. In AD research, GNN-based approaches have been applied to integrate molecular networks and neuroimaging features, facilitating biomarker discovery, disease classification, and mechanistic investigation [113,114].

More recently, transformer-based architectures have attracted increasing attention in AD-related applications due to their ability to model long-range dependencies through self-attention mechanisms. Originally developed for natural language processing (NLP), transformers have been adapted for clinical text analysis, speech representation learning, neuroimaging interpretation, and multimodal biomedical data analysis. In clinical NLP applications, transformer-based models can extract meaningful information from unstructured medical records and physician notes, supporting automated phenotyping and clinical decision assistance. Moreover, their flexible attention mechanisms enable the modeling of complex interactions among heterogeneous features, providing a foundation for advanced multimodal learning frameworks that integrate imaging, molecular, and clinical information.

**Figure 6:**
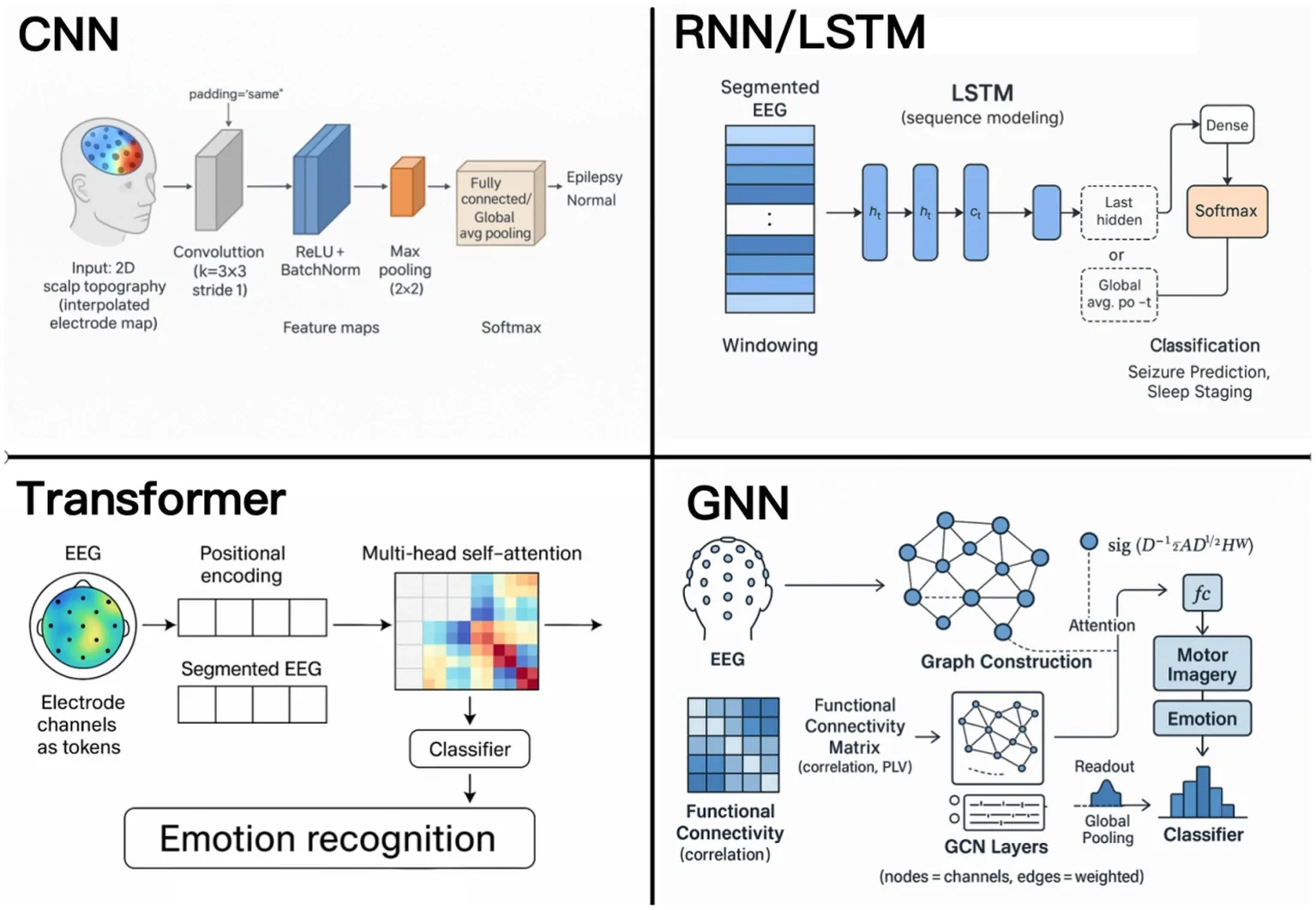
Representative deep learning architectures for EEG analysis, including CNNs, RNN/LSTM, Transformers, and GNNs, which model different representations of temporal and inter-channel relationships. Reproduced with permission from Ref. [115].

**Figure 7:**
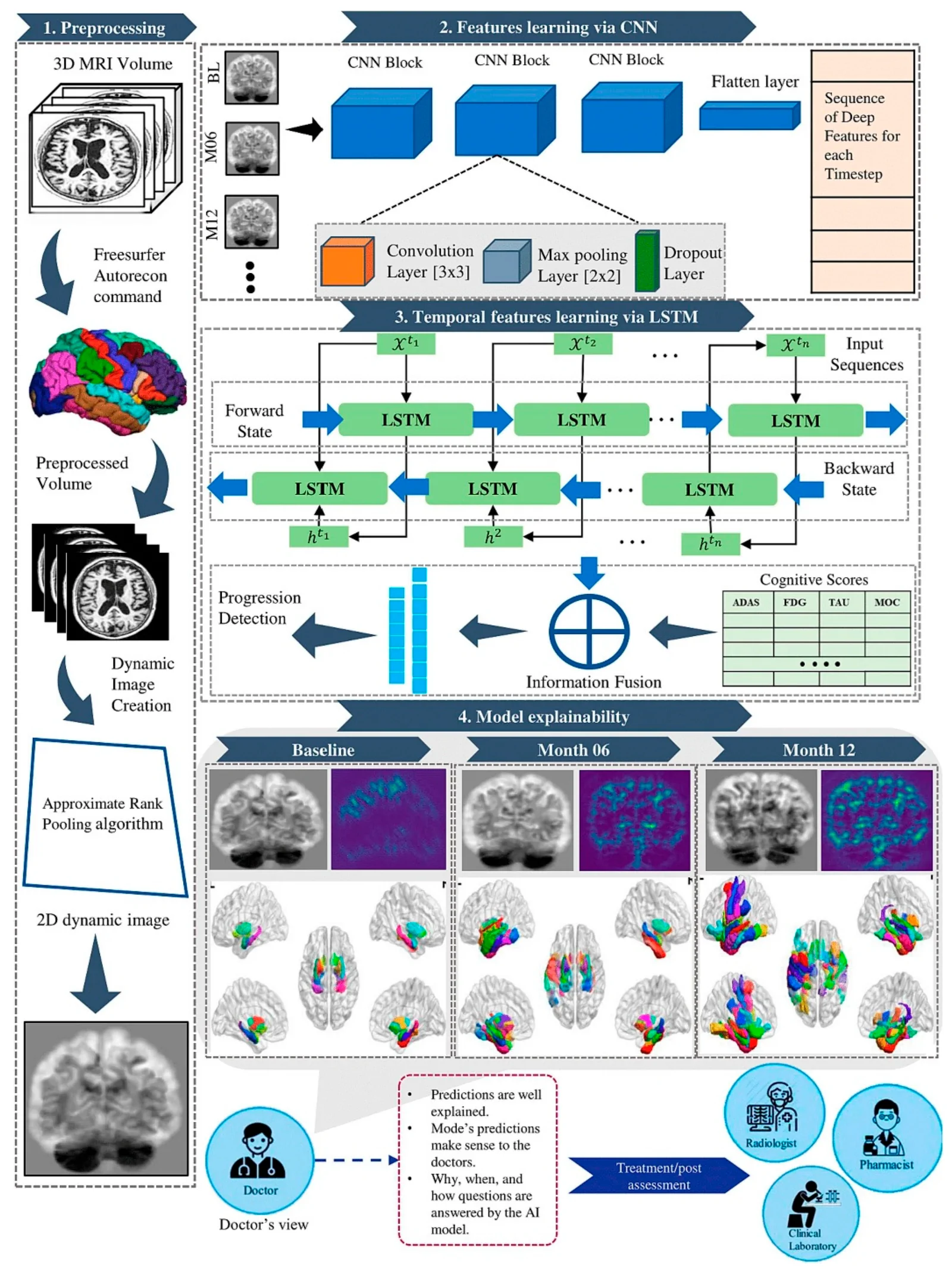
A hybrid CNN–BiLSTM framework for AD progression prediction using longitudinal MRI data, integrating spatial feature extraction and temporal sequence modeling. Reproduced with permission from Ref. [116].

Deep learning architectures are not inherently restricted to specific data modalities; instead, their suitability is largely determined by the intrinsic structure and characteristics of the input data. Different architectures are designed to capture distinct patterns, including spatial dependencies, temporal dynamics, sequential relationships, and complex graph structures. As illustrated in Figure 6, EEG signals can be modeled using various DL frameworks [115]. Beyond applying individual architectures, hybrid deep learning frameworks have also emerged to integrate complementary representation capabilities from multiple networks. For instance, in AD progression prediction, a CNN-BiLSTM framework was developed to extract spatial features from longitudinal MRI data using CNNs and subsequently model temporal changes in brain structures through bidirectional LSTM networks (Figure 7) [116]. Representative applications of DL across different data modalities, datasets, and tasks are summarized in Table 2.

### 3.3. Multimodal AI and Data Fusion Strategies

AD is a complex multifactorial disorder characterized by interactions among genetic susceptibility, molecular alterations, brain structural and functional changes, and clinical manifestations. Therefore, single-modality approaches often provide an incomplete representation of disease heterogeneity and may overlook complementary information contained in different biological levels. Multimodal AI approaches aim to integrate heterogeneous data sources, including neuroimaging, multi-omics, electrophysiological signals, clinical assessments, and behavioral measurements, to construct more comprehensive representations of AD-related alterations.

The integration strategy for multimodal data is typically categorized into early integration, intermediate integration, and late integration. Early integration combines feature sets from different modalities into a single input dataset, which is then processed by a unified model. This approach simplifies the model design process, as only one predictive model needs to be trained. However, early integration requires the model to simultaneously accommodate diverse data modalities, and differences in feature dimensionality, data distribution, and representation characteristics may introduce learning imbalances. Furthermore, directly combining heterogeneous modalities can increase feature complexity and introduce redundant or noisy information, necessitating feature selection or dimensionality reduction methods to mitigate the curse of dimensionality. Late integration trains separate models for individual modalities and subsequently combines their outputs to generate final predictions. This strategy enables the use of modality-specific models optimized for different data characteristics and is particularly suitable for heterogeneous datasets with different acquisition protocols or missing modalities. Additionally, late integration maintains flexibility by allowing new modalities to be incorporated without retraining the entire framework. However, because fusion occurs at the decision level, it may fail to capture complex interactions and complementary relationships among modalities, limiting the ability to fully exploit multimodal information. Intermediate integration transforms data from different modalities into modality-specific intermediate representations, which are subsequently integrated within hidden layers of the model during training. By propagating prediction errors through feature extraction and fusion layers, the model can optimize shared representations across modalities. This strategy is typically based on the assumption that heterogeneous modalities contain partially shared latent information, allowing the model to capture both modality-specific characteristics and cross-modal interactions [131,132,133,134].

**Figure 8:**
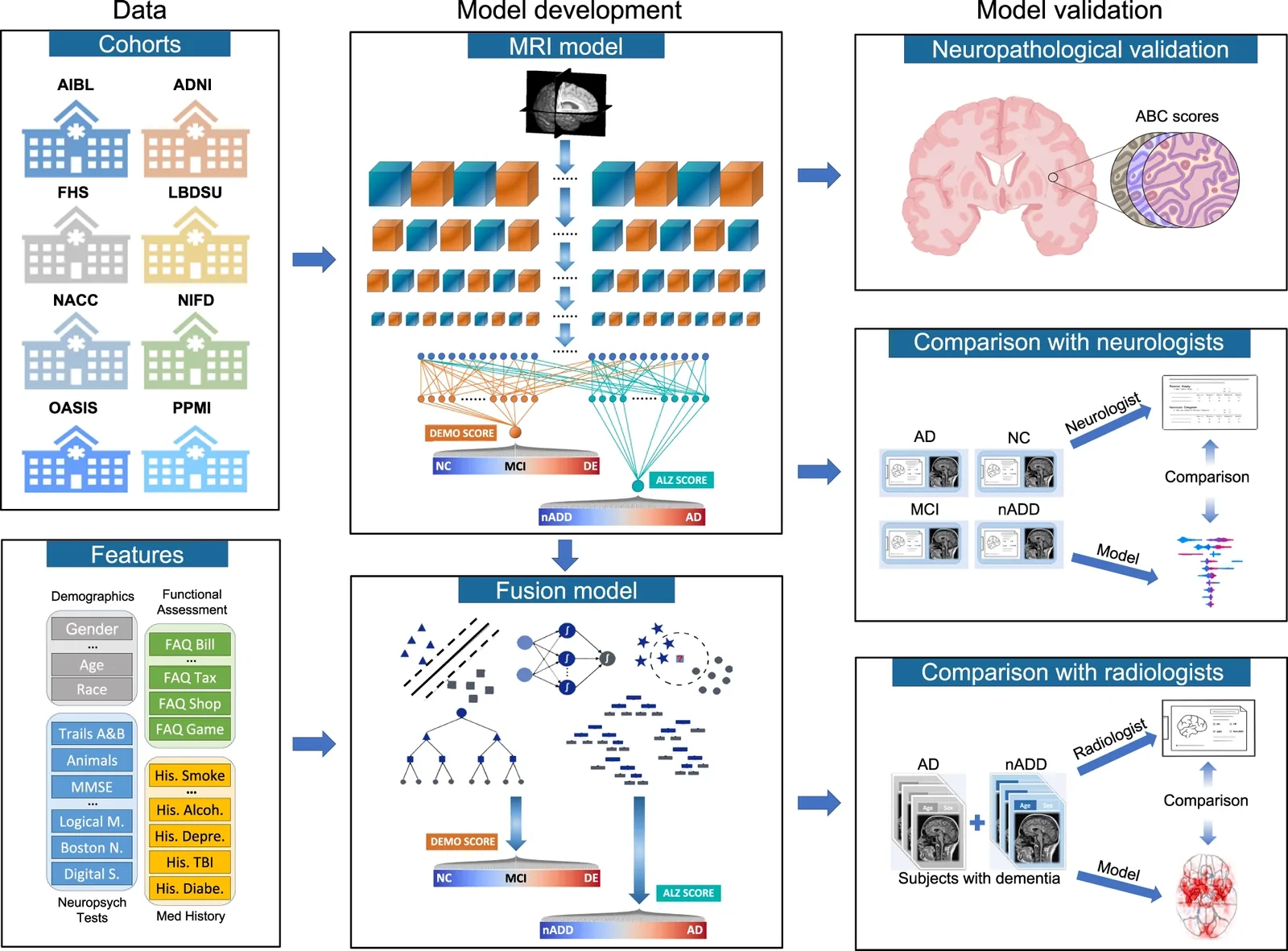
Multimodal deep learning framework integrating neuroimaging and clinical information for AD detection through modality-specific feature extraction and fusion. Reproduced with permission from Ref. [135].

**Figure 9:**
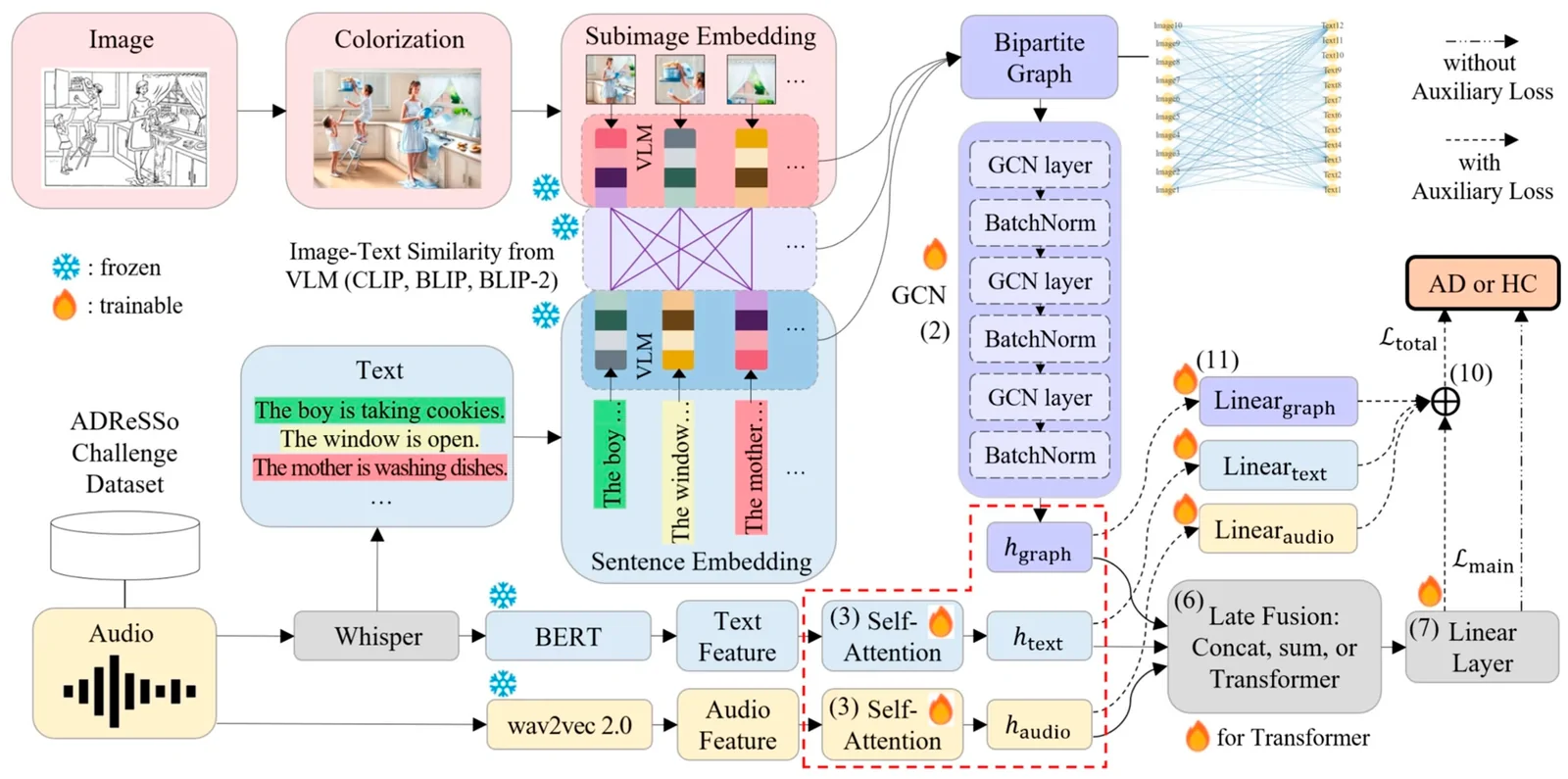
Attention-based multimodal fusion framework for AD recognition. Audio, text, and visual representations are integrated through cross-attention mechanisms to capture complementary interactions between heterogeneous modalities. Reproduced with permission from Ref. [137].

A typical multimodal deep learning architecture consists of modality-specific encoders, a fusion module, and a downstream prediction network. Modality-specific encoders are designed to capture unique characteristics within each data type, and the extracted representations are subsequently integrated through fusion modules to generate a unified multimodal representation for AD detection or prognosis prediction. As illustrated in Figure 8, multimodal frameworks can integrate complementary information from neuroimaging and non-imaging clinical variables through modality-specific learning branches and subsequent feature-level or decision-level fusion, enabling more comprehensive characterization of AD-related alterations [135]. Attention mechanisms have attracted increasing attention due to their ability to dynamically estimate the contribution of different modalities or features. Unlike simple feature concatenation, attention-based models can assign different weights to modality-specific information and capture interactions between heterogeneous data sources. For example, cross-attention mechanisms enable one modality to selectively attend to informative features from another modality, facilitating the identification of complementary relationships between molecular, imaging, and clinical characteristics. Such approach has demonstrated improved performance in AD classification [136]. A representative attention-based multimodal framework for AD recognition is illustrated in Figure 9, where modality-specific representations from audio, text, and visual information are aligned through cross-modal attention mechanisms to enhance complementary information integration [137]. Representative applications of multimodal AI across different data modalities, datasets, tasks, and fusion strategies are summarized in Table 3.

## 4. Entering the LLM Era: A Paradigm Shift for AD

The development of language-based artificial intelligence for AD has progressed from task-specific deep learning toward increasingly general-purpose foundation models. Pretrained language models such as BERT, RoBERTa, and ELECTRA advanced clinical language analysis by learning reusable contextual representations from large-scale corpora. However, these encoder-based models were generally adapted to predefined tasks through fine-tuning or task-specific classifiers, and thus remained largely within task-specific analytical frameworks. The emergence of generative LLMs, including GPT- and LLaMA-family models, has extended this paradigm beyond pretrained representation learning. Through prompting, instruction following, in-context learning, and natural-language generation, generative LLMs can support multiple language-based tasks within a common framework, shifting AI development from task-specific modeling toward more general-purpose and interactive systems. Accordingly, the distinction between pretrained language models and generative LLMs lies not simply in model scale, but in how models are used: pretrained encoders primarily provide representations for predefined tasks, whereas generative LLMs can flexibly understand, generate, and transform clinical information. This evolution is particularly relevant to AD, where clinically meaningful evidence is distributed across heterogeneous modalities, including speech, EHRs, neuroimaging, electrophysiological signals, and molecular biomarkers. Multimodal and knowledge-augmented LLMs further extend these capabilities by integrating non-textual data and external biomedical knowledge, creating opportunities for more comprehensive assessment and knowledge-assisted clinical reasoning. Thus, the potential paradigm shift lies in moving from narrowly defined predictive pipelines toward general-purpose, multimodal, and knowledge-augmented systems, although its clinical significance remains to be established through rigorous external and prospective validation. Representative applications of language model-based approaches across different data modalities, tasks, and model architectures are summarized in Table 4.

### 4.1. Language-Model-Based Approaches for Speech and Language-Based AD Assessment

Pretrained language models have been increasingly applied to dementia-related speech datasets, including the DementiaBank Pitt corpus and the Alzheimer’s Dementia Recognition through Spontaneous Speech (ADReSS) challenge. By learning contextual representations from speech transcripts, models such as BERT, RoBERTa, and ELECTRA can capture AD-associated linguistic characteristics, including reduced lexical diversity, semantic errors, repetitive expressions, and simplified sentence structures. Balagopalan et al. fine-tuned BERT on ADReSS transcripts and achieved 83.3% accuracy in distinguishing individuals with AD from cognitively normal individuals while simultaneously predicting MMSE scores [148]. Yuan et al. incorporated pause-related information into RoBERTa and improved classification performance on the Pitt corpus [149], and 89.6% accuracy on ADReSS using ERNIE-large with pause encoding [150], suggesting that combining linguistic content with speech-related characteristics may improve AD-related classification. Since speech abnormalities in AD involve both what patients say and how they speak, combining language representations with acoustic features can provide complementary diagnostic information. Altinok et al. developed a cross-attention framework integrating RoBERTa-based text representations with spectrogram features extracted from speech signals, achieving 90.1% accuracy on ADReSS [151]. Similarly, other multimodal approaches combined sentence-level language embeddings with acoustic representations derived from speech encoders, demonstrating that cross-modal attention mechanisms can effectively capture interactions between linguistic content and vocal characteristics [152,153].

More recently, generative LLMs have extended language-based approaches beyond conventional encoder-based classification and representation learning. LLMs have been explored as flexible tools for linguistic feature extraction, screening, and task-specific analysis through prompting or embedding extraction. Heitz et al. used GPT-4 to generate structured linguistic descriptors and achieved an AUC of 93.1% on ADReSS using random forest classification [154]. Agbavor and Liang extracted GPT-3 embeddings from speech transcripts and demonstrated their effectiveness for dementia screening compared with traditional acoustic features [155]. Furthermore, studies applying language-specific models to Mandarin and Slovak datasets indicate the potential of language-model-based approaches across different linguistic contexts, although performance remains influenced by dataset characteristics and language-specific adaptation [156,157]. However, many of these studies are based on relatively small and curated speech datasets, and their reported performance may be sensitive to dataset composition and evaluation settings. External and prospective validation remains limited.

### 4.2. Language-Model-Based Approaches for EHR Analysis and Clinical Information Extraction

Language models have been increasingly applied to EHR data for clinical information extraction and longitudinal risk assessment. An early example is AD-BERT, which learned representations from sequential clinical notes to predict MCI-to-AD conversion [158]. Using records from Northwestern Medicine Enterprise Data Warehouse (NMEDW) with external validation at Weill Cornell Medicine (WCM), AD-BERT outperformed seven baseline models, suggesting that pretrained language representations can capture temporal patterns associated with disease progression.

Building on advances in representation learning, one study assessed GPT-4 and LLaMA-2 on over 34,000 cognitive-related notes, with manual review of 765 selected notes. GPT-4 achieved 83% accuracy and 89.7% sensitivity for MMSE extraction, outperforming LLaMA-2 [159]. Beyond predefined variables, Shao et al. developed LLM-MINE, a large language model-based phenotype mining framework that used few-shot prompting and expert-defined phenotype lists to identify features associated with Alzheimer’s disease and related dementias (ADRD) from clinical notes in the MIMIC-IV database. The extracted phenotypes included memory impairment, language deficits, executive dysfunction, and behavioral abnormalities, and showed significant differences across cognitively normal, MCI, and ADRD groups. LLM-MINE also improved unsupervised disease staging compared with dictionary-based and biomedical named entity recognition approaches [160]. Beyond information extraction and phenotyping, LLMs have also been applied to harmonize clinical concepts across heterogeneous biomedical resources. PromptLink combined SapBERT-based embeddings for candidate retrieval with GPT-4-based reasoning and self-verification for concept normalization between EHRs and biomedical knowledge graphs [161]. Unlike rule-based or supervised approaches requiring manually curated terminologies, PromptLink demonstrated zero-shot generalizability using only concept names, facilitating the integration of EHRs with knowledge bases for downstream applications in AD research, including biomarker discovery and mechanistic investigation. Nevertheless, the evidence remains largely based on retrospective EHR datasets and task-specific evaluations, and the generalizability of these approaches across institutions and clinical settings remains to be established.

### 4.3. Multimodal and Knowledge-Augmented LLMs for AD Assessment

Advances in multimodal LLMs have provided new opportunities to bridge different modalities by aligning non-textual biomedical signals with language representations, enabling more comprehensive AD assessment and interpretable reasoning.

Several multimodal frameworks have combined neuroimaging with clinical information. BrainMIND-LLM integrates 3D MRI and PET images with demographic characteristics, cognitive assessments, CSF biomarkers, and APOE genotype to generate diagnostic reports. Using the ADNI dataset, BrainMIND-LLM achieved superior report-generation performance compared with general-purpose LLMs, demonstrating the potential of multimodal models for integrating heterogeneous clinical information and generating clinically interpretable outputs [162]. Similarly, Kiguchi et al. proposed a knowledge graph-based framework integrating MRI, gene expression, biomarkers, EEG, and clinical indicators from independent cohorts. LLMs were used to analyze relationships among multimodal features and generate biological hypotheses, revealing potential associations involving metabolic factors, neuroinflammation, tau pathology, EEG patterns, and gene expression [163]. Together, these studies illustrate how LLMs can facilitate the integration and interpretation of heterogeneous biomedical information beyond single-modality prediction.

Knowledge augmentation provides another direction for extending LLM-based AD assessment. AlzheimerRAG combines retrieval-augmented generation with multimodal information processing to integrate biomedical literature containing text, tables, and images. By retrieving relevant evidence from external knowledge sources, AlzheimerRAG improved performance on biomedical question-answering benchmarks while reducing unsupported responses [164]. Similarly, HoloDx incorporated knowledge injection and memory-based attention mechanisms to integrate clinical expertise with LLM representations, achieving improved classification accuracy and generalization across multiple AD datasets [165]. These approaches highlight the potential of combining multimodal patient information with external biomedical knowledge to support evidence-informed reasoning and more interactive forms of AD assessment. At present, most reported applications remain focused on model performance, information integration, or proof-of-concept demonstrations, with limited evidence from prospective and clinically representative evaluations. Their effectiveness and utility in routine clinical workflows therefore remain to be established.

## 5. Limitations and Future Perspectives

Although AI models have demonstrated promising performance in AD-related assessment, several challenges remain before these technologies can be widely translated into clinical practice.

A major challenge is the limited availability and heterogeneity of high-quality AD datasets. Compared with other fields with large-scale datasets, AD research is constrained by relatively small sample sizes, incomplete longitudinal follow-up, and the high cost of acquiring multimodal biological data, including neuroimaging, omics profiles, electrophysiological signals, and clinical records. Moreover, differences in data acquisition platforms, preprocessing procedures, and analytical pipelines introduce substantial variability across cohorts, limiting the generalizability of AI models. This issue is particularly prominent for multi-omics and multimodal approaches, where integrating heterogeneous data types across different biological levels remains challenging [166,167]. Future studies should promote multicenter collaboration, standardized data collection protocols, and large-scale data sharing to improve model robustness. In addition, many existing AD datasets lack sufficient representation of diverse populations, which may introduce algorithmic bias and restrict the applicability of AI systems across different ethnic and socioeconomic groups. Increasing population diversity in future datasets will be essential for developing equitable and clinically applicable AI models.

Understanding the decision-making process of AI algorithms is crucial for building trust among clinicians and patients. However, many ML and DL models remain difficult to interpret because of their complex architectures, limiting their ability to provide biological insights and reducing acceptance in clinical settings. Explainable AI approaches, including LIME, SHAP, attention visualization, and feature importance analysis, have been developed to improve model transparency. Nevertheless, generating biologically meaningful and clinically reliable explanations remains challenging. For LLM-based approaches, additional concerns arise because prompt-generated rationales may improve readability but do not necessarily reflect the actual reasoning process of the model. Moreover, attention-based interpretation methods do not always provide causal explanations [168,169,170]. Future AI systems should incorporate more rigorous interpretability frameworks and involve clinicians in evaluating the relevance of model outputs.

Reproducibility and reliability also remain important challenges. The lack of data and code sharing, as well as incomplete reporting of preprocessing procedures and model configurations, limits the ability to independently reproduce published findings. This issue is particularly relevant for deep learning models and LLM-based approaches, where results may be affected by hyperparameter selection, model initialization, prompt design, and evaluation strategies. In addition, LLMs introduce unique challenges, including hallucination, inconsistent outputs across different prompts, and difficulties in handling noisy real-world clinical data. Although retrieval-augmented generation and knowledge grounding approaches have been proposed to improve factual reliability, further validation is required before LLMs can be safely applied in clinical environments. Establishing standardized evaluation frameworks, external validation protocols, and transparent reporting guidelines will be critical for improving the reproducibility and clinical reliability of AI-driven AD research [171,172,173].

Regulatory and data governance considerations also represent important barriers to clinical translation. AI systems intended for clinical use may require compliance with applicable regulatory frameworks, particularly when they are used for diagnostic assessment or clinical decision support. At the same time, the use of sensitive clinical and biomedical data raises concerns regarding privacy, security, informed consent, interoperability, and responsible data sharing. Addressing these issues through appropriate regulatory oversight, robust data governance frameworks, and standardized clinical evaluation will be essential for the safe and responsible integration of AI and LLMs into clinical practice.

## 6. Conclusions

In summary, the rapid evolution of AI, from traditional ML and DL to emerging LLMs, has provided powerful tools for analyzing diverse and complex data sources in AD diagnosis. By enabling automated feature learning, multimodal data integration, and knowledge-guided interpretation, AI approaches offer new opportunities for improving early detection, disease characterization, and personalized clinical assessment of AD. However, several challenges remain before AI-based diagnostic systems can be widely implemented in clinical practice, including limited and heterogeneous datasets, insufficient model interpretability, concerns regarding reliability and reproducibility, and the need for rigorous clinical validation. Future advances will require interdisciplinary collaboration among AI researchers, clinicians, and biomedical scientists to develop accurate, transparent, and clinically applicable AI systems that can effectively support precision diagnosis and management of AD.

## Figures and Tables

**Table 2:** Summary of representative deep learning-based studies for Alzheimer’s disease.

Study	Task	Dataset	Input	Method	Result			
					Acc	Auc	Sen	Spe
[117]	CN vs. AD	ADNI (CN: 162; pMCI: 76; sMCI: 134; AD: 137)	sMRI	CNN	84	92	-	-
	pMCI vs. CN				79	83	-	-
	sMCI vs. pMCI				62	59	-	-
[118]	CN vs. AD	ADNI (CN: 100; pMCI: 76; sMCI: 128; AD: 93)	sMRI, PET	CNN-LSTM	94.8	97.1	97.7	92.5
	pMCI vs. CN				86.4	91.1	84.9	88.8
	sMCI vs. CN				63.4	69.2	70.6	59.6
[119]	CN vs. AD	ADNI (CN: 34; eMCI: 40; lMCI: 40; AD: 34)	fMRI	CNN-RNN	94.5	-	-	-
	eMCI vs. lMCI				61.8	-	-	-
[120]	CN vs. AD	ADNI (CN: 102; MCI: 97; AD: 94)	PET	3D CNN	89.2	-	87.2	91.2
	MCI vs. AD				71.7	-	70.2	73.2
	CN vs. MCI				62.3		59.8	64.7
[121]	AD vs. MCI vs. CN	Brazil (CN: 52; MCI: 37; AD: 52)	EEG	AlexNet	98.9	-	-	-
[122]	CN vs. AD	Private (CN: 24; AD: 24)	EEG	LSTM	91	1	100	83
				CNN	93	95	86	100
				ANN	74	84	62	86
				CNN-LSTM	93	97	86	100
[123]	CN vs. MCI	Private (CN: 16; MCI: 11)	EEG	ResNet	92	-	-	-
[124]	CN vs. AD	Private (CN: 20; AD: 20)	EEG	GNN	92	98.4	97.4	86.7
[125]	CN vs. AD	OASIS (CN: 660; AD: 155)	fMRI, external knowledge	GNN	77.8	60	-	-
	CN vs. MCI	ADNI (CN: 186; AD: 154)			66.2	61	-	-
[126]	CN vs. AD	GEO (CN: 74; AD: 68)	Genomic, Epigenomic	DBN	99.6	-	-	-
[127]	CN vs. AD	ROSMAP (CN: 169; AD: 182)	Transcriptomic, Epigenomic	MORE	82.9	90.3	-	-
				MOGONET	81.4	88.2	-	-
				MOGLAM	81.6	88.5	-	-
				MOADLN	81.6	88.6	-	-
[128]	CN vs. AD	TalkBank (CN: 94; AD: 166)	Transcript/Text	AWD-LSTM	81.5	-	-	-
[129]	CN vs. AD	Private (CN: 40; MCI: 40; AD:40)	Text	GCNN	91	96	91	90
	CN vs. MCI				79	83	78	80
[130]	CN vs. AD	ADRC (CN: 10; AD: 9)	SERS spectra	CNN	92	-	-	-
[77]	CN vs. AD	Private (CN: 5; AD: 20)	FET biosensor	CNN	100	-	-	-

**Table 3:** Summary of representative multimodal AI-based studies for Alzheimer’s disease.

Study	Task	Dataset	Input	Method	Fusion	Result			
						Acc	Auc	Sen	Spe
[138]	CN vs. AD	OASIS (CN: 605; AD: 493)	sMRI, FDG-PET	CNN	Intermediate	95	93	93.3	96.7
[137]	CN vs. AD	ADReSS (CN: 115; AD: 122)	Image, audio, text	GCN	Intermediate + late	87.9	-	94.4	-
[139]	CN vs. AD	DementiaBank Pitt (CN: 243; AD: 257)	Audio, text	GNN	Intermediate	84.8	-	-	-
[140]	CN vs. AD	ADNI (CN: 122; AD: 88)	sMRI, FDG-PET	CNN + attention	Intermediate	98.1	98.4	95.8	96.8
[141]	CN vs. AD	ADNI (CN: 364; AD: 214)	sMRI, SNP	AlzCLIP	Late	81	80	83	79
		UKB (CN: 200; AD: 61)				79	81	82	76
[142]	CN vs. MCI vs. AD	ADNI (CN: 97; MCI: 43; AD: 10)	sMRI, gene, plasma	RF	Early	-	95	-	-
[143]	CN vs. AD	ADNI (213 CN: 213; AD: 222)	fMRI, SNP	Residual Cross-modal Attention + GAT	Intermediate	95.7	-	-	-
[144]	CN vs. AD	CMUH (CN: 83; AD: 60)	EEG, SNP	XGBoost	Intermediate	72.7	72.4	-	72.4
				RF		78.9	78.8	-	77.2
				SVM		92.1	91.6	-	95.1
[145]	CN vs. MCI vs. AD	ADNI (total: 410)	sMRI, EEG, gene	Multi-View Encoder + Cross-Attention Fusion + Progression Graph	Intermediate	94.3	97.5	-	-
	sMCI vs. pMCI					93.7	96.8	-	-
[146]	CN vs. MCI vs. AD	Private (CN: 83; MCI: 145; AD: 112)	sMRI, text report, structured report	VGG16 + LSTM + attention + KNN	Early	79.6	-	-	-
[147]	CN vs. MCI	BEGAD (CN: 37; MCI: 20; AD:21)	Audio, eye-tracking data	FNN	Intermediate	81.5	76.5	-	-
	CN vs. AD					85.9	80.5	-	-

**Table 4:** Summary of representative language model-based studies for Alzheimer’s disease.

Study	Task	Dataset	Input	Method	Result
[148]	CN vs. AD MMSE score regression	ADReSS (CN: 78; AD: 78)	Spontaneous speech transcripts, audio	Feature-based (509 features + SVM) vs. fine-tuned BERT	Classification: BERT: Acc 83.3, F1 83.3; SVM Acc 81.3, F1 81.2 Regression: Ridge RMSE 4.56 (MAE 3.50); BERT numerically outperforms SVM but difference not significant (*p* > 0.05)
[149]	CN vs. AD	DementiaBank Pitt (CN: 309; AD: 243)	Spontaneous speech transcripts, pause duration encoding	BERT/RoBERTa re-trained on pause-encoded transcripts; pause augmentation	AugPauseRoBERTa Acc 85.4 PauseRoBERT Acc 83.3 RoBERTa Acc 82.3
[150]	CN vs. AD	ADReSS (CN: 78; AD: 78)	Spontaneous speech transcripts, pause encoding	BERT/ERNIE fine-tuning + pause encoding + ensemble	ERNIE-large + 3 pauses: Acc 89.6 ERNIE0p: Acc 85.4 BERT3p: Acc 85.4 BERT0p: Acc 81.3
[151]	CN vs. AD	ADReSSo (CN: 115; AD: 122)	Speech audio, Whisper-transcribed text	ViT + RoBERTa + cross-attention fusion	Cross-attention: Acc 90.1 F1 90.1 Shallow fusion: Acc 87.3 F1 87; BERT + ViT + Gated Acc 90.1 F1 89 Dual BERT: Acc 82 F1 82 Multimodal BERT-ViT: Acc 79 F1 81 BERT + ViT + Co-Attention: Acc 87 F1 86
[152]	CN vs. AD	DementiaBank (CN: 89; AD: 168); ADReSS (CN: 78; AD: 78); IVA (CN: 62; AD:29)	Spontaneous speech, manual transcripts	TSAC-ATT: Acoustic: wav2vec2.0 + TSAC; Linguistic: BERT	TSAC-ATT: DementiaBank: Acc 81.5, F1 81.5; IVA: Acc 88.6, F1 88.2; ADReSS: Acc 81.3, F1 81.2 Linguistic-only: DementiaBank: Acc 81.8, F1 81.8; IVA: Acc 86.4, F1 86; ADReSS: Acc 77.1, F1 77.1
[153]	CN vs. MCI	Self-collected NTU-AM-MM (CN: 237; MCI: 127)	Spontaneous speech, manual transcripts	Dual-Modal: Acoustic: eGeMAPS + TabNet + Transformer Encoder; Linguistic: BERT + speaker embedding + conversation embedding	Dual-Modal: Acc 78, F1 77, Recall 79, Pre 84, Spe 82 Linguistic-only: Acc 72, F1 72, Recall 72, Pre 75, Spe 72 Acoustic-only: Acc 72, F1 69, Recall 64, Pre 84, Spe 81
[154]	CN vs. AD	ADReSS (CN: 78; AD: 78)	Transcribed speech	GPT-4 extracted 5 semantic features + established linguistic features + Random Forest	AUC 93.1; outperforms established features alone (88.5) and fine-tuned GPT-4 (88.6)
[155]	CN vs. AD	ADReSSo (CN: 78; AD: 78)	Transcribed speech	GPT-3 text embeddings + ML classifiers	Acc 80.3, F1 80, AUC 89; outperforms acoustic features (Acc 68.1, AUC 69)
[156]	CN vs. AD (Cross-domain recognition)	NCMMSC2021 (Chinese; CN: 44, AD: 26); ADReSS (English; CN: 78; AD: 78); Ivanova (Spanish; CN: 197; AD: 74)	Transcribed speech	GPT-3 embeddings + RF-MDSA + SVM	Single corpus: CN Acc 74.6, F1 73.4; EN Acc 82.4, F1 78.7; SP Acc 62.8, F1 61.8 Cross-domain Acc: EN + SP→CN 71.8, CN + SP→EN 72.9, CN + EN→SP 56.1
[157]	AD vs. no-AD	EWA-DB (Slovak; 285 speakers; 5 pictures; CN: 209; MCI:36; AD: 25)	Transcribed Slovak speech (picture descriptions)	BERT fine-tuning (SlovakBERT, mBERT, XLM-RoBERTa, RemBERT, LaBSE)	Best Avg F1: SlovakBERT 74, LaBSE 74, mBERT 69, XLM-RoBERTa 69, RemBERT 69
[158]	MCI-to-AD progression prediction	NMEDW (3657 MCI: 396 progressors, 3261 non-progressors); WCM (2563 MCI: 548 progressors, 2015 non-progressors)	Unstructured EHR notes	AD-BERT (pre-trained on AD notes)	NMEDW: AUC 84.9/F1 44 (no-restrict), 78.5/39.3 (6-mo), 72.8/45.4 (1-yr), 69.9/55.2 (2-yr) WCM: AUC 88.3/68, 68.6/26.9, 66.6/37.2, 64.4/53.9;
[159]	Cognitive exam info extraction	NYU Langone (34,465 notes; 722 manually reviewed; 309 double-reviewed)	Unstructured EHR notes	GPT-4 + LLaMA-2	ChatGPT: MMSE Acc 83.0, Sen 89.7, CDR Acc 87.1, Sen 84.3% LLaMA-2: MMSE Acc 66.4%, Sen 69.9; Inter-rater agreement (Fleiss‘ κ): 0.94 for MMSE, 0.89 for CDR
[160]	ADRD phenotype extraction	MIMIC-IV (CN: 8372; MCI: 841; ADRD: 8327)	Unstructured clinical notes (Discharge notes)	LLM-MINE (gemma-3-12b-it + few-shot prompting)	Clustering ARI = 0.290, NMI = 0.232 (K = 2), significantly outperforms dictionary-based and NER baselines; phenotype prevalence differs significantly across CN/MCI/ADRD (*p* < 0.001)
[161]	Biomedical concept linking (EHR concepts → KG concepts)	MIID (MIMIC-III → iBKH): 1493 pairs; CISE (CRADLE → iBKH): 1500 pairs	EHR concept names	PromptLink (SapBERT candidate retrieval + GPT-4 two-stage prompting + self-verification)	Acc: 77.56 (MIID), 88.8 (CISE)
[162]	Clinical report generation	ADNI (CN: 235; MCI: 326; AD: 184)	sMRI, FDG-PET, clinical data	BrainMIND-LLM (3D ViT + Q-Former + T5)	BERTScore 0.913, ROUGE-L 0.507, BLEU-4 0.177, METEOR 0.471
[163]	Multi-modal data integration and hypothesis generation	ADNI, Kaggle (total AD: 1045; CN: 1622; MCI: 89	MRI, EEG, biomarkers, clinical data, gene expression	Knowledge graph + LLMs (ChatGPT 4o, Claude 3.5 Sonnet, Gemini 2.0 Flash)	Identified metabolic-inflammatory-tau pathway (r > 0.6, *p* < 0.001) & EEG-gene correlations (r = 0.42–0.58, *p* < 0.01); 5 consensus hypotheses; cross-validation variance < 15%
[164]	Multimodal RAG for AD literature QA	PubMed (2000 AD articles); BioASQ, PubMedQA	User queries; PubMed literature (text, tables, images)	AlzheimerRAG (LLaMA-2-7b-PubMed + LLaVA + cross-modal attention)	Recall 0.88, Precision@10 85, F1 86
[165]	CN vs. AD	ADNI (CN: 724; AD:519)	sMRI, clinical text, domain knowledge (LLM-generated + expert)	HoloDx (3D ViT + Longformer + Knowledge Injection + Memory)	Acc 93.3, Auc 91.8, Sen 88.7, Spe 95
		AIBL (CN: 361; AD:81)			Acc 91.8, Auc 89.4, Sen 96.6, Spe 80.6
		RENJI (CN: 30; AD: 180)			Acc 94.7, Auc 94.8, Sen 93.8, Spe 95.8

## Data Availability

No new data were created or analyzed in this study. Data sharing is not applicable to this article.
